# Quantitative analysis of correlation between AT and GC biases among bacterial genomes

**DOI:** 10.1371/journal.pone.0171408

**Published:** 2017-02-03

**Authors:** Ge Zhang, Feng Gao

**Affiliations:** 1 Department of Physics, Tianjin University, Tianjin, China; 2 Key Laboratory of Systems Bioengineering, Ministry of Education, Tianjin University, Tianjin, China; 3 SynBio Research Platform, Collaborative Innovation Center of Chemical Science and Engineering (Tianjin), Tianjin University, Tianjin, China; Beijing Institute of Genomics Chinese Academy of Sciences, CHINA

## Abstract

Due to different replication mechanisms between the leading and lagging strands, nucleotide composition asymmetries widely exist in bacterial genomes. A general consideration reveals that the leading strand is enriched in Guanine (G) and Thymine (T), and the lagging strand shows richness in Adenine (A) and Cytosine (C). However, some bacteria like *Bacillus subtilis* have been discovered composing more A than T in the leading strand. To investigate the difference, we analyze the nucleotide asymmetry from the aspect of AT and GC bias correlations. In this study, we propose a windowless method, the Z-curve Correlation Coefficient (ZCC) index, based on the Z-curve method, and analyzed more than 2000 bacterial genomes. We find that the majority of bacteria reveal negative correlations between AT and GC biases, while most genomes in Firmicutes and Tenericutes have positive ZCC indexes. The presence of PolC, purine asymmetry and stronger genes preference in the leading strand are not confined to Firmicutes, but also likely to happen in other phyla dominated by positive ZCC indexes. This method also provides a new insight into other relevant features like aerobism, and can be applied to analyze the correlation between RY (Purine and Pyrimidine) and MK (Amino and Keto) bias and so on.

## Introduction

According to Chargaff’s second parity rule, bases tend to share equal percentages in the scale of whole DNA strand, i.e., Adenine (A) = Thymine (T) and Guanine (G) = Cytosine (C), only under an ideal circumstance without mutation or selection [1]. In reality, nucleotide composition asymmetries widely exist in most bacterial genomes.

It is usually considered that the cause of nucleotide asymmetries is the joint effects of mutational pressure and selective pressure [2]. Asymmetries on mutational frequency, DNA repair efficiency [3], as well as an excess of deamination of cytosine to thymine in the coding strand during the transcription process [4, 5], would lead to asymmetric mutational pressure between the leading and lagging strands. As for selective pressure, a preference in the third codon position for G over C and T over A, and the unequal distribution of coding regions have been revealed between the leading and lagging strands [4, 6]. This theory explains the asymmetric condition of the majority of genomes, which the leading strand is rich in G and T, while the lagging strand is rich in A and C. However, some genomes reveal different asymmetric patterns. For example, *B*. *subtilis* (Firmicutes) is rich in A and G in the leading strand, which is regarded as purine asymmetry (PAS) [7]. Besides, a strong gene distribution bias between the leading and the lagging strands, which is named as strand-biased gene distribution (SGD) [8], as well as the presence or absence of PolC are also considered unique to genomes in Firmicutes [9].

In order to investigate why there exist different nucleotide asymmetric patterns, whether there are certain correlation between AT and GC biases, whether the features of PolC, PAS and strong SGD are only confined to the phylum Firmicutes, and are there any other genomic features related, we analyzed the nucleotide bias with more than 2000 bacterial genomes.

There are three major approaches measuring nucleotide asymmetries. The first approach is GC asymmetry, which can be calculated by (C-G)/(C+G) [10]. G and C represent the frequency of occurrence of the equivalent base in a particular sequence in a defined length. This method adopts a window sliding strategy to calculate C-G deviations through a genome. However, the major weakness of this method is its window-size dependent property. Plots with small-sized windows may be less illustrative because of the visible fluctuations, while larger windows will hide precise details like polarity switches. The second approach is referred to as Cumulative GC skew (CGS skew), a sum of (G-C)/(G+C) in adjacent windows from an arbitrary start to a given point in a sequence [11]. This improvement strengthens the polarity switches and increases the visibility. Taking *Mycoplasma*. *pneumoniae* [12] as an example, the cumulative method readily reveals polarity switches in the CGS skew plot while in the GC skew plot is much harder to be detected. The third approach is the Z-curve method [13]. Different from previous methods, the Z-curve method is a geometrical approach to genome analysis without sliding window. Another advantage of the Z-curve is its intuitiveness, enabling global and local compositional features of genomes to be grasped quickly in a perceivable form. The methodology of the Z-curve is suitable platform on which other methods, such as statistics, can be integrated to address bioinformatics questions [13].

We find that plenty of research has been carried out on GC bias but less on AT bias. Besides, few studies have focused on exploring the relationship between AT and GC biases. Here, we propose a quantitative index, the Z-curve Correlation Coefficient (ZCC) index, measuring the correlation between AT and GC biases based on the Z-curve method. There are several reasons for choosing the Z-curve method. Firstly, the Z-curve method is a windowless technique, whose results are extremely precise, and Z-curve as a three-dimensional curve that uniquely represents the given DNA sequence contains all details of the sequence. Then, the correlation coefficient index based on the Z-curve can reflect the real correlation between AT and GC biases along genomes at the single nucleotide level [14]. Secondly, the three parameters represent the excess of purine over pyrimidine, keto over amino, and weak hydrogen bonds to strong hydrogen bonds, respectively, which can be easily applied on exploring relationships of other features and making comparisons. Thirdly, the geometrical method offers intuitive figures, which reveal disparity trends, polarity switches between the leading and lagging strands as well as other genomic features.

We hypothesized that different nucleotide asymmetric patterns would be reflected in the signs and numerical values of ZCC indexes. Bacterial genomes in the phyla dominated by positive ZCC indexes might also reveal PAS and strong SGD like Firmicutes. Besides, PolC might also appear in other phyla than Firmicutes. In this study, we analyzed 2187 bacterial genomes to test these hypotheses and reached the conclusions related to the nucleotide bias correlation.

## Materials and methods

### Datasets

In this study, we retrieved the information of 2187 bacteria from the DoriC database (http://tubic.tju.edu.cn/doric/). DoriC is a database of *oriC*s (replication origins) in bacteria and archaea [15, 16], which can be downloaded from http://tubic.tju.edu.cn/doric/download.php. The genome files and the annotation files of these bacteria were obtained from NCBI (ftp://ftp.ncbi.nlm.nih.gov/genomes/refseq/bacteria/).

Polymerase information was accessed from the supplementary data of a survey and summary article at NAR Online [17]. C-family polymerases are clearly partitioned into PolC and DnaE with high resolution in phylogenetic tree, while DnaE polymerase are further divided into 4 groups, i.e. DnaE1, DnaE2, DnaE3, and DnaEX for the rest [17]. Considering the lack of obvious boundaries between each DnaE polymerase groups, and that detailed classification may introduce complexity to conclusions, we united different types of DnaEs (DnaE1-DnaE3 and DnaEX) as DnaE in general. We referred to the Gene ID Conversion Tool in DAVID [18] for genome matching and acquired 772 bacteria in DoriC with corresponding DNA polymerase information. Polymerase information including but not limited to putative DNA polymerase combination and species names has been collected in the S1 Table.

### The ZCC index definition

The Z-curve method is a 3-dimensional curve determined with the following parameters:

$$x_{n}=(A_{n}+G_{n})-(C_{n}+T_{n})$$

$$y_{n}=(A_{n}+C_{n})-(G_{n}+T_{n})$$

$$z_{n}=(A_{n}+T_{n})-(C_{n}+G_{n})$$

where *n =* 0, 1, 2, …, *N*. *A*_*n*_, *C*_*n*_, *G*_*n*_, *T*_*n*_ are accumulated occurrence numbers of A, C, G, T, counting from an arbitrary base to the *n*th base along a sequence with length *N*. *x*_*n*_, *y*_*n*_, *z*_*n*_ represent the excess of purine over pyrimidine, keto over amino and weak hydrogen bonds over strong hydrogen bonds, respectively. The AT and GC disparity curves are defined by (*x*_*n*_*+y*_*n*_)/2 and (*x*_*n*_*-y*_*n*_)/2, which calculate the excess of A over T and G over C, along a genome respectively.

The definition of Pearson Correlation Coefficient *r* is as follows,

$$r=\frac{\sum_{i=1}^{n}\left(a_{i}-\bar{a})(b_{i}-\bar{b}\right)}{\sqrt{\sum_{i=i}^{n}{\left(a_{i}-\bar{a}\right)}^{2}\sum_{i=i}^{n}{\left(b_{i}-\bar{b}\right)}^{2}}}.$$

According to the principle of the Z-curve method, we appoint AT disparity curves, (*x*_*i*_*+y*_*i*_)/2 as *a*_*i*_, and GC disparity curves, (*x*_*i*_*-y*_*i*_)/2 as *b*_*i*_. *x*_*i*_ and *y*_*i*_ are two parameters of the Z-curve, both of which accumulate from 0 to *i*. *n* denotes the genome length.

## Results

### The signs of ZCC index values

**Fig 1:**
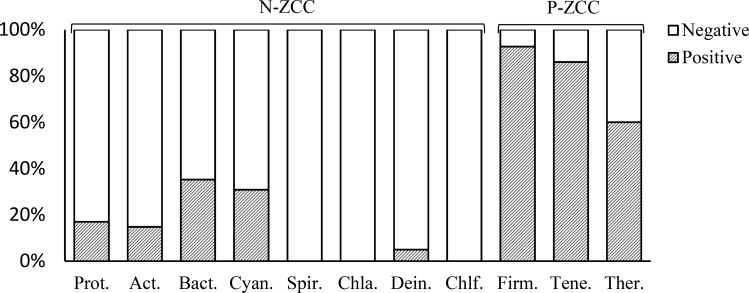
Percentages of bacterial genomes with the positive and negative ZCC indexes in 11 phyla. The abbreviated phylum names of each histogram represent the full names of Proteobacteria, Actinobacteria, Bacteroidetes, Cyanobacteria, Spirochaetes, Chlamydiae, Deinococcus-Thermus, Chloroflexi, Firmicutes, Tenericutes and Thermotogae, respectively. We classified phyla Proteobacteria, Actinobacteria, Bacteroidetes, Cyanobacteria, Spirochaetes, Chlamydiae, Deinococcus-Thermus and Chloroflexi as the Negative ZCC phylum group (N-ZCC group), while phyla Firmicutes, Tenericutes and Thermotogae are classified as the Positive ZCC phylum group (P-ZCC group), according to the predominant signs of genomes in the corresponding phylum.

Based on the definition of ZCC index, the sign of its value represents general correlation trends between AT and GC biases along genomes. To inquire the hypothesis whether there exist genomes in other phyla revealing similar genomic features with Firmicutes, we grouped genomes by phylum. Considering the randomness of small samples, we only presented the results of the phyla with more than 15 species to draw a reliable conclusion. Consequently, there are 11 phyla with more than 15 species counting 2115 species among all 2187 bacteria in DoriC, which are Proteobacteria, Firmicutes, Actinobacteria, Bacteroidetes, Cyanobacteria, Spirochaetes, Chlamydiae, Tenericutes, Deinococcus-Thermus, Chloroflexi and Thermotogae. For convenience, brief information of bacterial genomes in these 11 phyla is summarized in Table 1. Percentages of bacterial genomes with the positive and negative ZCC indexes in these 11 phyla are also shown in Fig 1. In general, 64.5 percent of bacteria among datasets have negative ZCC indexes. However, we noticed that predominant signs of ZCC indexes differ from phyla. Bacterial genomes in the phyla of Firmicutes and Tenericutes are notably positively correlated, taking percentages of 92.7 and 86.0 respectively. The phylum Thermotogae is also rich in positive signed genomes (60.0%). On the contrary, genomes of the rest 8 phyla are negative-dominated, among which genomes in phyla Spirochaetes, Chlamydiae and Chloroflexi are entirely negative. Therefore, we classified these phyla into two groups: the Positive ZCC phylum group (P-ZCC group) and the Negative ZCC phylum group (N-ZCC group). The grouping results are determined by the sign the majority of genomes reveal in the corresponding phylum. Among these 11 phyla, we classified Firmicutes, Tenericutes and Thermotogae as the P-ZCC group, while Proteobacteria, Actinobacteria, Bacteroidetes, Cyanobacteria, Spirochaetes, Chlamydiae, Deinococcus-Thermus and Chloroflexi are classified as the N-ZCC group.

**Table 1:** Summary information of ZCC indexes in different phyla.

Phylum	Average ZCC index	Standard Deviation	Number of Bacterial Genomes		
			Negative ZCC index	Positive ZCC index	Total
Proteobacteria	-0.594	0.567	875 (82.9%)	180 (17.1%)	1055
Actinobacteria	-0.617	0.475	195 (85.2%)	34 (14.8%)	229
Bacteroidetes	-0.288	0.657	55 (64.7%)	30 (35.3%)	85
Cyanobacteria	-0.350	0.595	47 (69.1%)	21 (30.9%)	68
Spirochaetes	-0.848	0.195	57 (100.0%)	0	57
Chlamydiae	-0.941	0.043	52 (100.0%)	0	52
Deinococcus-Thermus	-0.782	0.332	19 (95.0%)	1 (5.0%)	20
Chloroflexi	-0.897	0.131	17 (100.0%)	0	17
Firmicutes	0.818	0.415	34 (7.3%)	433 (92.7%)	467
Tenericutes	0.663	0.505	7 (14.0%)	43 (86.0%)	50
Thermotogae	0.096	0.634	6 (40.0%)	9 (60.0%)	15
Sum	-	-	1364 (64.5%)	751 (35.5%)	2115

### The numerical values of ZCC index

**Fig 2:**
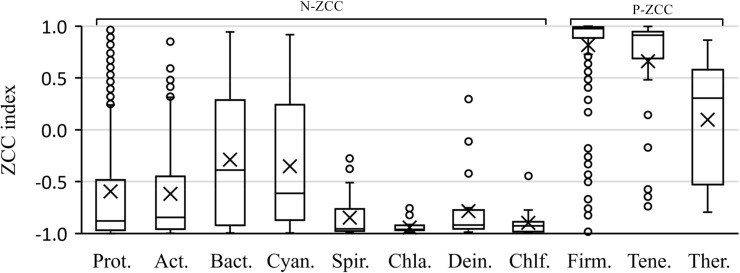
The box-plot of ZCC indexes in different phyla. Small rings represent outliers with extreme ZCC indexes. The genomes tending to have large absolute values of ZCC indexes indicate the correlation between AT and GC disparities are widely and obviously exist.

Besides the correlation trends revealed by the signs of ZCC indexes, numerical values of ZCC indexes reveal the correlation degree between AT and GC biases. Mean values and corresponding standard deviations of each phylum are listed in Table 1. We found that the signs of mean values perfectly match the aforementioned grouping results, namely P-ZCC group and N-ZCC group. A boxplot depicting ZCC indexes of 2115 genomes grouped by phylum are graphed in Fig 2. Small circles in Fig 2 represent outliers with extreme ZCC indexes, whose values are greater than Q3+1.5IQR or less than Q1-1.5IQR. Q1, Q3 and IQR represent upper quartile, lower quartile and quartile range respectively. As shown in the box-plot, the majority of genomes tend to have large absolute values of ZCC indexes. Medians of most phyla are larger than 0.8, and some are even close to 1. Genomes in the same phyla tend to have large absolute values except phyla Bacteroidetes, Cyanobacteria, Thermotogae and few outlier genomes. These observations suggest that correlations between AT and GC biases are widely and obviously exist.

**Fig 3:**
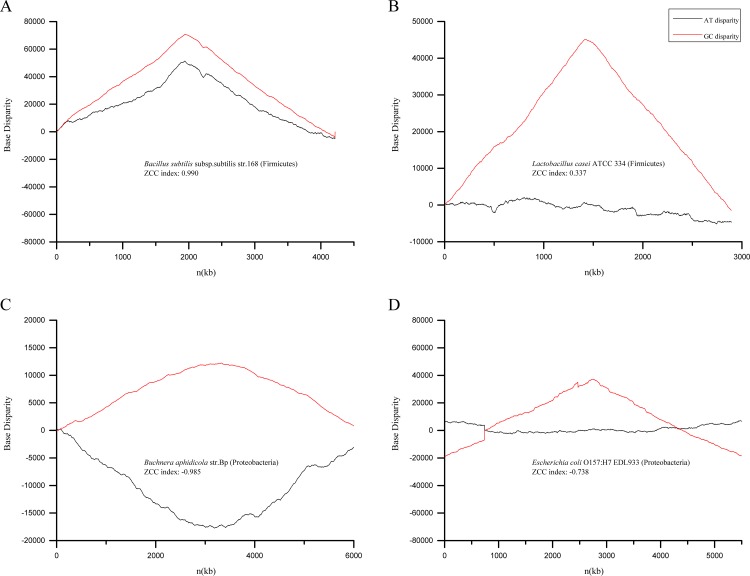
The Z-curve disparity figures. Among different genomes, GC disparity curves always show inverted-V curves, while the shapes of AT disparity curves vary from phyla, ZCC index signs and numerical values.

The Z-curve method also brings in an intuitive figure measuring nucleotide biases. We plotted the AT and GC disparity curves of several genomes, which start from replication origins (Fig 3). GC disparity curves always show inverted-V shapes reversing at positions near half-lengths. However, shapes of AT disparity curves vary from phyla, ZCC index signs and numerical values. AT disparity curves of genomes with large absolute values of ZCC indexes reveal consistent or contract trends with GC disparity curves, and reverse at same paces. Genomes with relative small absolute values of ZCC indexes show indistinctive trends in AT disparity curves (Fig 3B and 3D). Multiple asymmetric patterns between AT and GC disparity curves suggest that the transcription or replication mechanisms of genomes might vary from phyla.

### Polymerase distribution

DNA polymerase III is responsible for the replication of bacterial genomes, and plays an important role in driving sequence variations. Researchers have found that all known bacterial genomes encode one or more DNA polymerases without a single exception [17]. The core enzyme of DNA polymerase III for bacteria is the α-subunit. There are two basic classes of α-subunit genes: DnaE and PolC. Among 772 genomes with polymerase information, all encode one or more DnaEs, while PolC are not always present. Considering that PolC always co-occurs with either DnaE1 or DnaE3, we classified genomes with PolC to the PC group regardless of how many DnaEs they accompanied. And the rest genomes are classified to the DE group. Genome distributions are listed in Table 2. The front numbers represent genome numbers in each phylum among 772 genomes. We found that distributions of polymerase groups are well separated to different phyla. Genomes in the PC group also tend to belong to P-ZCC phyla with only a single exception in Actinobacteria, while genomes in the DE group are rich in N-ZCC phyla with few exceptions in Firmicutes. Then we removed outliers detected in Fig 2, and new distribution results are listed behind the slash in Table 2. Separating distributions of DE and PC groups between N-ZCC and P-ZCC phyla, in this way, are extremely remarkable with only a single exception. Explanations for exceptional bacterial genomes are discussed in the Discussion section.

**Table 2:** Genome distributions to DE and PC groups in different phyla.

Polymerase group	Prot.	Act.	Bact.	Cyan.	Spir.	Chla.	Dein.	Chlf.	Firm.^a^	Tene.^a^	Ther.^a^
DE^b^	321/285^d^	101/93	66/66	23/23	17/16	8/7	12/10	10/9	7 ^e^ /1^e^	0/0	0/0
PC^c^	0/0	1 ^e^ /0	0/0	0/0	0/0	0/0	0/0	0/0	153/137	17/15	12/12

^a^ Represent the Positive ZCC phyla. The rest phyla without superscript belong to the Negative ZCC phyla.

^b^ DE represent genomes which only encode DnaE1-DnaEXs without PolC.

^c^ PC represent genomes which encode PolC as well as DnaE1-DnaEXs.

^d^ Each cell contains two numbers, X/Y. X represents the genome number among all 772 datasets. Y represents the genome number eliminating outliers in Fig 2.

^e^ Exceptional genome numbers compared with the general trend.

### Multiple features related to the ZCC grouping rule

**Fig 4:**
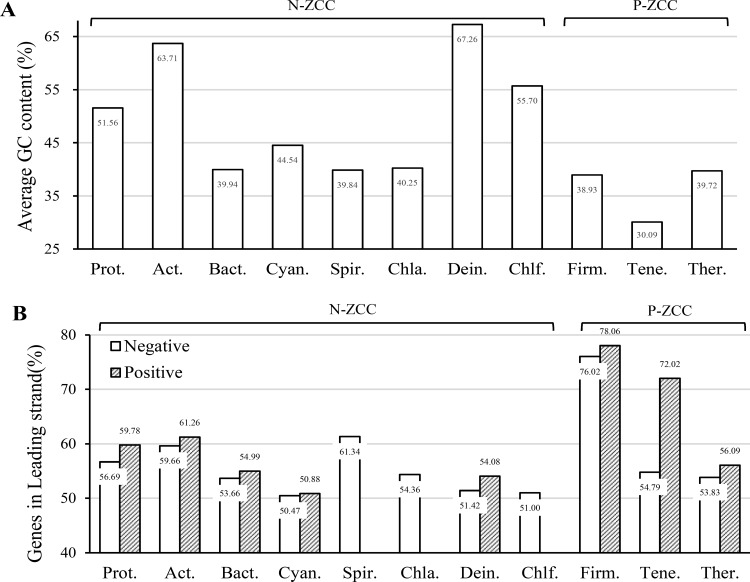
**(A) Mean values of GC contents of genomes in each phylum. (B) Average percentages of genes in the leading strand grouped by genomes with the positive and negative ZCC indexes in each phylum.** In the histogram (A), mean values of GC content in N-ZCC phyla are entirely larger than those in P-ZCC phyla. The histogram (B) shows that genes are preferred to located in leading strands. Besides, the degree of strand-biased gene distribution (SGD) is generally stronger among genomes with positive ZCC indexes than those with negative ZCC indexes.

Besides DNA polymerases, many other genomic properties also reveal correlations with the ZCC grouping rules for bacterial phyla. For examples, Fig 4A shows that mean values of GC contents in N-ZCC phyla are entirely larger than those in P-ZCC phyla. In Fig 4B supports the previous findings that genes prefer to locate in leading strands. Then, genomes in each phylum are further sorted by individual ZCC index signs, and genomes in Spirochaetes, Chlamydiae and Chloroflexi have no species with positive ZCC indexes. Among the rest phyla in Fig 4B, the degree of strand-biased gene distribution (SGD) is stronger among genomes with positive ZCC indexes than those with negative ZCC indexes. Detailed information including genome lengths and GC contents is available in the S1 Table.

## Discussion

The Z-curve method has been proved as an effective technique detecting asymmetrical nucleotide distributions around replication origins [13]. Maximum and minimum values around the switches of its disparity curves can be approximatively regarded as the replication origin and terminus respectively [19], and the leading and lagging strands can thus be determined. On the other hand, the deamination of C to T in the leading strand causes a universal phenomenon of G enrichment in the leading strand. Therefore, genomes with positive and large ZCC indexes can be identified having G and A enrichment in the leading strand, which is regarded as purine asymmetry (PAS) [7]. Strand-biased gene distribution (SGD) happens under the selection process to avoid frequent polymerase collisions [20]. Co-oriented collisions occur for genes in the leading strand and head-on collisions occur for genes in the lagging strand. Localization of genes in the leading strand will reduce the collision rate because head-on collisions occur more frequently than co-oriented collisions [21]. It has been found that PolC is responsible for the synthesis of the leading strand [22]. The existence of PolC will accelerate the replication process in the leading strand, explaining the phenomena of stronger SGD in genomes with positive ZCC indexes in Fig 4B. On the other hand, purine-rich genes prefer to locate in the leading strand, because the purine-richness can prevent nonspecific RNA-RNA interactions and excessive formation of double-stranded RNA [23]. The existence of PolC also accelerates genes exchanges and causes purine richness in the leading strand.

Previous studies have proved that the presence of PolC was correlated with PAS and stronger SGD, and all these features were unique to genomes in Firmicutes [8]. Accordingly, Hu *et al* classified genomes into the Phylum Firmicutes group (F group) and the non-Phylum Firmicutes group (NF group) [7], and proved that PolC plays an essential role driving PAS of genomes in the F group. At the same time, they noticed that partial genomes in the phylum Tenericutes and Thermotogae hold similar PAS features with genomes in the F group. In the present study, Firmicutes, Tenericutes and Thermotogae are classified as the same group. In Fig 4, the phylum Tenericutes even reveals the lowest GC content and the most significant differences of average SGD between the genomes with positive and negative ZCC indexes. We find that all Tenericutes genomes in our study belong to the order Mollicutes. According to phylogenetic analysis, Mollicutes was previously thought to be a class within Firmicutes [24]. Later on the basis of its unique phenotypic properties such as the lack of rigid cell walls and other evidences, Mollicutes was thus placed under a new phylum called Tenericutes [24]. As for Thermotogae, the exact position of Thermotogae within the phylogenetic tree is not clear yet because different markers have yielded varying results and a significant degree of horizontal acquisition of genes from other species has made the situation even more confusing [25, 26]. The phylum Fusobacteria, whose ribosomal molecular phylogeny and core genome contents indicate a lineage branching out at the base of Firmicutes [8], turn out to have 40% genomes in our study with positive ZCC indexes. These cases indicate that the presence of PolC, PAS and stronger SGD are not exclusive in the Firmicutes. And the minority Firmicutes genomes which lack PAS and PolC are presumed to have lost the gene encoding PolC [27], considering the fact that bacterial genomes are highly dynamic in nature and they are continuously undergoing the processes of gene loss and gain [28].

The ZCC index is a quantitative and intuitive method measuring the nucleotide bias from the aspect of AT and GC correlation. This method can classify the phyla of bacteria into the positive and negative groups according to the signs of corresponding ZCC indexes. Bacterial genomes with positive ZCC indexes usually have coherent genomic features like lower GC content, stronger SGD and the presence of PolC, which is also consistent with the previous findings about the correlation between PAS, stronger SGD and PolC [7]. However, these features are not only confined to the phylum Firmicutes but also other phyla dominated by positive ZCC indexes. Comprehensive analysis on genomes with the same signs of ZCC indexes can also shed new light on phylogenetic studies. Besides, this method brings in a new perspective discovering more relevant features, like the RY (Purine and Pyrimidine) and MK (Amino and Keto) disparities, the genome length, the aerobism and so on. To sum up, the ZCC index is an effective method for nucleotide bias studies.

## Supporting information

S1 Table Detailed information of 2187 bacterial genomes. The table collects information of all bacterial genomes in this study, including ZCC indexes and DNA polymerase distributions. Many other genomic properties, like RY-MK Correlation indexes, aerobism and genome lengths have also been listed in the S1 Table. Updated information is also available at the website: http://tubic.tju.edu.cn/zcc/.
(XLSX)
